# A hemizygous mutation in the *FOXP3* gene (IPEX syndrome) resulting in recurrent X-linked fetal hydrops: a case report

**DOI:** 10.1186/s12920-021-00901-6

**Published:** 2021-02-26

**Authors:** Panicos Shangaris, Alison Ho, Andreas Marnerides, Simi George, Mudher AlAdnani, Shu Yau, Mattias Jansson, Jacqueline Hoyle, Joo Wook Ahn, Sian Ellard, Melita Irving, Diana Wellesley, Dharmintra Pasupathy, Muriel Holder-Espinasse

**Affiliations:** 1 Department of Women and Children’s Health, School of Life Course Sciences, Faculty of Life Sciences and Medicine, King’s College London, 10th Floor North Wing, St Thomas’ Hospital, Westminster Bridge Road, London, SE1 7EH UK; 2 Department of Clinical Genetics, Guy’s and St Thomas’ NHS Foundation Trust, London, UK; 3 Department of Molecular Genetics, Royal Devon & Exeter Hospital, Barrack Road, Exeter, EX2 5DW UK; 4 Wessex Clinical Genetics Service, Princess Anne Hospital, Southampton, SO16 5YA UK; 5 Department of Histopathology, St Thomas Hospital, Westminster Bridge Road, London, SE17EH UK; 6 Discipline of Obstetrics, Gynaecology and Neonatology, Westmead Clinical School, Faculty of Medicine and Health, University of Sydney, Sydney, Australia

**Keywords:** IPEX syndrome, *FOXP3*, Fetal hydrops, In utero transfusion

## Abstract

**Background:**

Fetal hydrops is excessive extravasation of fluid into the third space in a fetus, which could be due to a wide differential of underlying pathology. IPEX (immune dysregulation, polyendocrinopathy, enteropathy, X-linked) syndrome primarily affects males. It is a monogenic primary immunodeficiency syndrome of X-linked recessive inheritance due to *FOXP3* gene variants. It is characterised by the development of multiple autoimmune disorders in affected individuals.

**Case presentation:**

We present a rare cause of male fetal hydrops in the context of IPEX syndrome and discuss *FOXP3* gene variants as a differential for ‘unexplained’ fetal hydrops that may present after the first trimester.

**Discussion and conclusions:**

In all similar cases, the pathological process begins during intrauterine life. Furthermore, there are no survivors described. Consequently, this variant should be considered as a severe one, associated with intrauterine life onset and fatal course, i.e., the most severe IPEX phenotype.

## Background

The immune dysregulation-polyendocrinopathy-enteropathy x-linked (IPEX) syndrome is a primary immunodeficiency caused by variants in the *FOXP3* gene [1, 2]. *FOXP3* is a key control gene, which encodes a transcription factor regulating the development and function of regulatory T Cells (Tregs) [3]. Tregs are responsible for maintaining tolerance to self-antigens by suppressing the activity of autoreactive T cells that have escaped from the thymus [4]. IPEX syndrome can present antenatally, in the early neonatal period or later in life. The syndrome usually presents with severe enteropathy, chronic dermatitis, diabetes type 1, hypoparathyroidism and other immune-related issues [2]. Male patients typically die within the first two years of life if untreated. The only available, proven, treatment is allogeneic haematopoietic stem cell transplantation [1].

## Case presentation

**Fig. 1:**
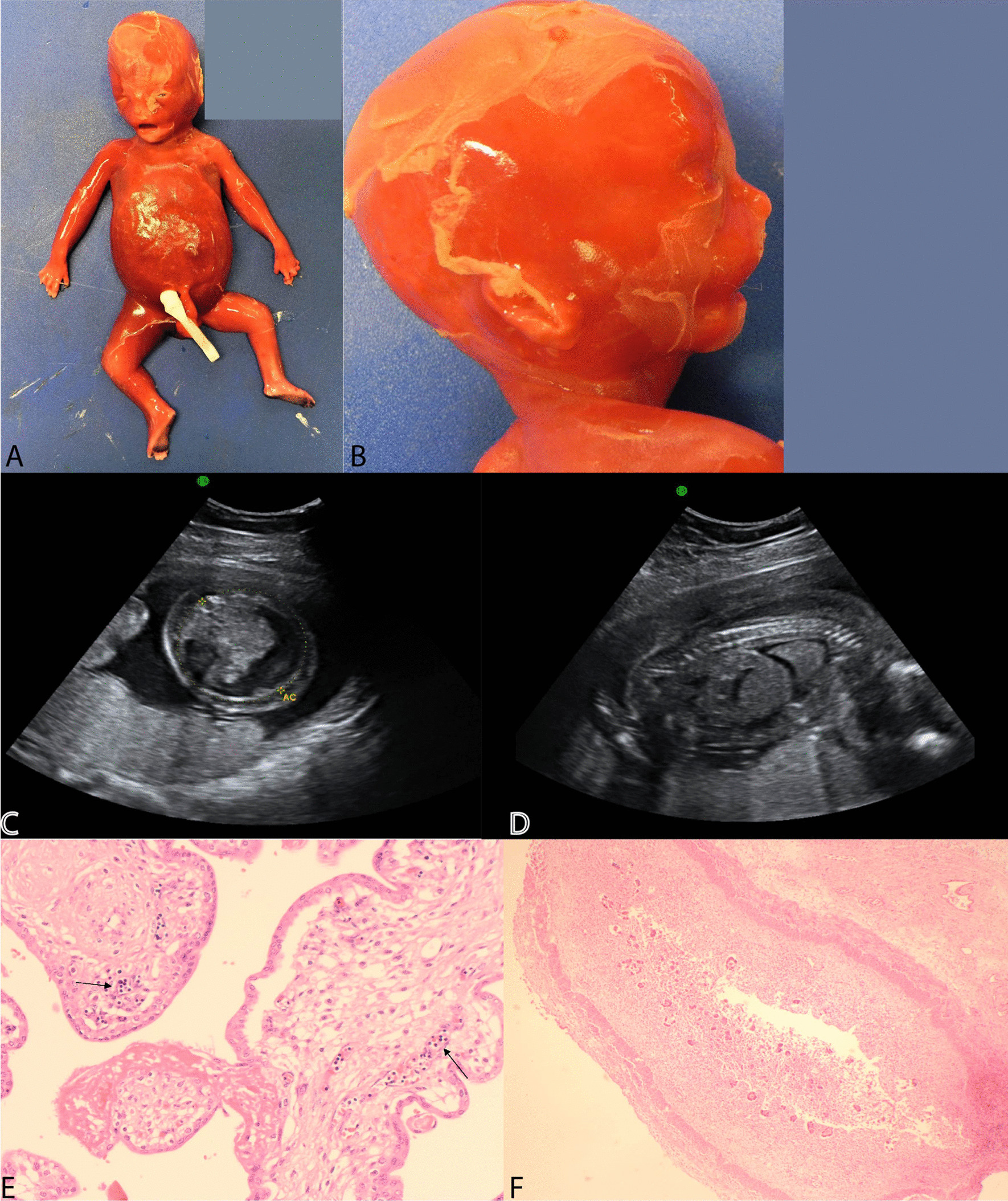
IPEX Fetus legend. **a** Macerated hydropic male fetus. **b** Profile picture of a hydropic fetus with a low set, posteriorly rotated ears and micrognathia. **c** Transverse image of the abdomen showing severe ascites. **d**. Sagittal image of the affected fetus showing hydrops fetalis. **e** H&E picture showing increased nucleated red cells in fetal vessels of chorionic villi (arrows). **f** H&E picture showing autolysed intestines with no significant pathology

A 28 year old Caucasian female in her first pregnancy with a low-risk quadruple test, underwent an anomaly ultrasound at 20 weeks’ and  6 days  of gestation, revealing significant fetal hydrops (Fig. 1c, d). She had an unremarkable past medical history, no family history of note and no recent viral illness. Talipes of the right foot was the only structural abnormality noted. Growth and liquor volume were normal; however, the MCA peak systolic velocity was above 1.5 Multiple of Median (MoM).

Fetal blood sampling was performed, which identified anaemia (Hb 60 g/L). In utero blood transfusion was performed (15 ml); however, towards the end of the procedure, fetal demise occurred. Post-natal investigations were negative for red cell antibodies, thrombophilia screen and systemic lupus erythematosus. There was no evidence of fetal infection in the amniotic fluid. A hydropic male baby with intrauterine growth restriction (Fig. 1a), low-set ears, micrognathia (Fig. 1b), a right-sided talipes and malrotated intestines, was revealed at post mortem examination. The placenta’s weight was above 90th centile with no evidence of abnormal trophoblast proliferation, mesenchymal dysplasia or storage disease. Oedematous villi, increased nucleated haematopoietic cells and decidual vasculopathy were identified (Fig. 1e). The pancreas, duodenum, small and large intestines were normally formed and focally autolysed on histology with no enteritis or intra-luminal inflammatory exudate (Fig. 1f).

Comparative genomic hybridisation (CGH) array analysis of DNA extracted from a rib using oligonucleotide arrays with ~60,000 probes across the genome was performed, and results were consistent with a normal male chromosome complement.

In her second pregnancy three years later, with a different partner, nuchal ultrasound was normal, and the first-trimester screening was ‘low risk’. Booking blood tests were negative for antibody and haemoglobinopathy screening. Subsequent serial ultrasound scans at 12, 16 and 18 weeks’ of gestation were normal; however, at 20 weeks’ and  3 days of gestation, a recurrence of fetal hydrops was detected. No sonographic evidence of fetal anaemia was present, and both growth velocity and amniotic fluid index were normal. No structural abnormalities were identified. The patient was well with no history of recent illness. Subsequent repeat thrombophilia screen, complement C3, complement C4, anticardiolipin and lupus anticoagulant antibody were all normal, as was a fetal infection screen. Simpson-Golabi-Behmel syndrome was excluded through *GPC3* gene molecular analysis on DNA extracted from the first fetus.

The patient declined invasive testing. She was managed expectantly with serial ultrasound scans and monitoring for maternal mirror syndrome. At 23 weeks’ and 3 days of gestation, fetal movements were reduced, and contractures of both wrists and feet were identified. Acetylcholine receptor antibodies and IgG subsets were normal. At 25 weeks’ and 3 days of gestation this progressed to bilateral talipes and persistent arm flexion. There was no evidence of fetal anaemia by MCA PSV doppler or abnormality of fetal echocardiography, but hydropic features had increased with hepatosplenomegaly. Given the rapid progression of fetal hydrops and poor prognosis, the couple opted for medical interruption of pregnancy.

A post mortem confirmed fetal hydrops with growth parameters in keeping with gestational age. The liver was enlarged, and two small accessory spleens were noted. The spleen, pancreas, large and small intestine showed extensive autolysis macroscopically and microscopically, not allowing any detailed histological assessment. These fetuses usually have CD3+ lymphocytic interstitial infiltration, CD4+ and CD8+ cells in the pancreatic parenchyma, as previously reported by Da Silva et al. [5].

There were no other abnormalities and no features suggestive of fetal anaemia. Virology of ascitic fluid, brain, heart, lung, liver and spleen tissue were negative. Antibody screen was negative. Array CGH showed a normal male chromosome complement.

Recurrence of fetal hydrops in two male pregnancies with different partners raised the suspicion of an X-linked condition. Linkage analysis showed that both babies had inherited the same maternal X chromosome. Whole exome sequencing was performed using Agilent SureSelectXT Clinical Research Exome library on an Illumina HiSeq 2500. The data was processed using an in-house pipeline with subsequent filtering and assessment of variants in QIAGEN Ingenuity Variant Analysis and identified a pathogenic heterozygous missense variant in exon 12 of the *FOXP3* gene [NM_014009.3:c.1189C > T p.(Arg397Trp)] on the maternal DNA. Sanger sequencing confirmed this result. Both fetuses were tested and found to be hemizygous for this variant, confirming a diagnosis of IPEX syndrome. The patient subsequently, had a successful pregnancy. She had prenatal testing with CVS, which showed a male fetus not affected by the condition.

## Discussion and conclusions

In the context of male fetal hydrops, variants in the *FOXP3* gene and resultant IPEX syndrome may be a cause for ‘unexplained’ cases following routine investigations. Other known in utero presentations include severe intrauterine growth restriction, fetal akinesia and recurrent male miscarriages [5–7]. Fetal hydrops may appear after the first trimester [6–9]. Early onset autoimmune haemolytic anemia is probably the cause of fetal hydrops in IPEX, so we could argue that IPEX-associated hydrops should be considered an immune process [10, 11].

This report reinforces that IPEX should be considered as an underlying disease in recurrent male miscarriages as well as in unexplained fetal hydrops. In addition, this is the 6th report on fetal-onset IPEX over a two-year interval, which suggests that this defect is more frequent than previously estimated. Until 2015 only post-natal onset cases were recognised. In addition to previous reports [5–8], there is a recently published paper with two additional cases of fetal-onset IPEX [9].

The variant described in this case report was previously identified by Xavier-da-Silva et al. [5] in a Brazilian family in a fetus presenting with hydrops at 27 weeks’ gestation and in a term newborn who developed diabetes mellitus during the first hours of life. The only other family with the same variant was reported by Levy-Lahad & Wildin [12], who described three siblings from the USA, presenting with IPEX manifestations at birth. One of them was a preterm infant. All three individuals with the *FOXP3* gene variant [c.1189C > T, p.(Arg397Trp)] in exon 12, had IPEX manifestations during the fetal period or on the 1st day of life. Thus, in all cases, the pathological process begins during intrauterine life. Furthermore, there are no survivors described. Consequently, this variant should be considered as a severe one, associated with intrauterine life onset and fatal course, i.e., the most severe IPEX phenotype.

This is in contrast to other causes of fetal hydrops (such as chromosomal abnormality [13]) and may be due to compromised ability to develop self-tolerance, as the fetus is significantly challenged by its endogenous immune system in the second trimester. Post mortems have revealed lethal in utero reactive T cell infiltration of multiple organs, plurivisceral inflammation and the presence of Charcot-Leyden crystals [7].

IPEX syndrome is a rare monogenic primary immunodeficiency syndrome of X-linked recessive inheritance [4]. The incidence and carrier status of IPEX syndrome is unknown. IPEX syndrome (OMIM 304790) is due to variants in the *FOXP3* gene mapped at Xp11.23, encoding a 431 amino acid transcriptional regulator protein forkhead box p3. FOXP3 is required to establish and maintain tolerance to self-antigens through the development and maintenance of CD4+, CD25+, Foxp3+ regulatory T cells [6].

Carrier females of IPEX syndrome are healthy, however, affected males typically present with neonatal-onset autoimmune endocrinopathies, enteropathies and dermatitis. Autoimmune phenomena include type 1 diabetes mellitus, pancytopenia, thyroid and renal disease. Immunodeficiency increases susceptibility to fatal infections. This constellation of pathologies leads to a poor prognosis and lethality, often as a neonate or within the first three years of life [6]. Current therapy for IPEX syndrome is supportive [14]. Immunosuppressive agents often fail due to toxicity and susceptibility to fatal infections. Haematopoietic stem cell transplant presents a potential management strategy [15]. The murine, equivalent to IPEX syndrome, is called a scurfy mouse. These mice are deficient in regulatory T cells. Manipulation of the scurfin protein, which is encoded by the *FOXP3* gene, might prevent or treat IPEX [16, 17].

IPEX syndrome patients are usually uneventful antenatally and appear phenotypically normal at birth; however, cases of fetal presentation are emerging. Intrauterine disease onset has been described in four families, presenting in the form of severe intrauterine growth restriction, fetal akinesia [6] with progressive fetal hydrops [5, 7, 8] and recurrent male miscarriages [5–7] before the second trimester.

Heightened awareness of IPEX syndrome as a differential for fetal hydrops is crucial for counselling of future pregnancies with a 50% risk of any subsequent male fetus being affected if the mother is a carrier. Carriers of IPEX syndrome are asymptomatic; thus, cascade screening is relevant to the patient’s relatives. They should be offered testing in the contest of non-invasive or invasive prenatal testing as well as preimplantation genetic diagnosis and subsequent IVF.

## Data Availability

The details of the variant analysed during the current study are available in the ClinVar repository, under the accession number SCV001468505. The raw datasets generated during the current study are not publicly available because it is possible that individual privacy could be compromised. It is possible to apply for permission to obtain access to the raw sequencing data and the details of the postmortem examination through the corresponding author.

## Abbreviations

IPEX: Immune dysregulation, polyendocrinopathy, enteropathy, X-linked
FOXP3: Forkhead box P3
Tregs: Regulatory T Cells
Hb: Haemoglobin
CGH: Comparative genomic hybridisation
DNA: Deoxyribonucleic acid
MCA PSV: Middle cerebral artery peak systolic velocity
IVF: In vitro fertilisation
